# Cold atmospheric plasma can effectively disinfect SARS‐CoV‐2 in the wastewater

**DOI:** 10.1002/EXP.20230012

**Published:** 2023-11-30

**Authors:** Hongbo Qin, Hengju Qiu, Ke Liu, Bixia Hong, Yuchen Liu, Chun Li, Mengzhe Li, Xiaoping An, Lihua Song, Eric Robert, Yigang Tong, Huahao Fan, Ruixue Wang

**Affiliations:** ^1^ College of Mechanical and Electrical Engineering Beijing University of Chemical Technology Beijing China; ^2^ College of Life Science and Technology Beijing University of Chemical Technology Beijing China; ^3^ University of Orléans Orléans France; ^4^ Beijing Advanced Innovation Center for Soft Matter Science and Engineering Beijing University of Chemical Technology Beijing China

**Keywords:** cold atmospheric plasma, coronavirus, reactive species, SARS‐CoV‐2, wastewater

## Abstract

COVID‐19 is currently pandemic and the detection of SARS‐CoV‐2 variants in wastewater is causing widespread concern. Herein, cold atmospheric plasma (CAP) is proposed as a novel wastewater disinfection technology that effectively inactivates SARS‐CoV‐2 transcription‐ and replication‐competent virus‐like particles, coronavirus GX_P2V, pseudotyped SARS‐CoV‐2 variants, and porcine epidemic diarrhoea virus in a large volume of water within 180 s (inhibition rate > 99%). Further, CAP disinfection did not adversely affect the viability of various human cell lines. It is identified that CAP produced peroxynitrite (ONOO^−^), ozone (O_3_), superoxide anion radicals (O_2_^−^), and hydrogen peroxide (H_2_O_2_) as the major active substances for coronavirus disinfection. Investigation of the mechanism showed that active substances not only reacted with the coronavirus spike protein and affected its infectivity, but also destroyed the nucleocapsid protein and genome, thus affecting virus replication. This method provides an efficient and environmentally friendly strategy for the elimination of SARS‐CoV‐2 and other coronaviruses from wastewater.

## Graphical abstract

Cold atmospheric plasma can produce peroxynitrite (ONOO^−^), ozone (O_3_), superoxide anion radicals (O_2_^−^), and hydrogen peroxide (H_2_O_2_) as the major reactive species to inactivate coronaviruses by disrupting the viral Spike protein and genomic RNA, thus hindering viral entry and replication. This study highlights the potential of cold atmospheric plasma as a promising approach to combat coronavirus infections.

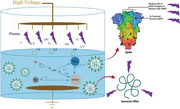

## 1 INTRODUCTION

Waterborne diseases pose a major threat to global public health. Approximately 80% of the wastewater worldwide is discharged into the environment without adequate treatment, causing serious pollution.^[^ ^1^ ^]^ Currently, a variety of viruses and bacteria such as enteroviruses, adenoviruses, and *Vibrio cholerae* have been detected in wastewater and drinking water.^[^ ^2^, ^3^ ^]^ Pathogens can survive for long periods in an aqueous environment and may be infectious, even in a highly diluted state.^[^ ^4^ ^]^ SARS‐CoV and norovirus in sewer pipes or sewage have caused outbreaks of waterborne disease.^[^ ^5^, ^6^, ^7^ ^]^ These pathogens pose a serious threat to human health.

SARS‐CoV‐2, the cause of the COVID‐19 pandemic, has been detected in wastewater in multiple countries.^[^ ^8^, ^9^, ^10^, ^11^ ^]^ Faeces are considered the main source of SARS‐CoV‐2 RNA detected in wastewater.^[^ ^12^, ^13^ ^]^ In addition, sputum, urine, and other excrement from patients can cause viral contamination as they enter sewers. With the global spread of the highly transmissible SARS‐CoV‐2 Omicron variant, the frequency of viral RNA detected in hospital wastewater (HWW) has increased.^[^ ^14^ ^]^ Multiple researchers have monitored SARS‐CoV‐2 in wastewater and reported that most contaminated wastewater irrigation plants had viral loads in the range of 10^4_^‐10^6^ copies/L, with the highest value of 10^8^ copies L^‐1^ in India.^[^ ^15^ ^]^ In addition to the detection of virus in wastewater, previous studies have demonstrated the successful isolation of infectious SARS‐CoV‐2 from the faeces of COVID‐19, indicating the potential risk of faecal–oral transmission.^[^ ^16^ ^]^ Additionally, during the SARS‐CoV epidemic outbreak in 2003, a large number of people were infected in a neighbourhood in Hong Kong, China, and one study found that multiple SARS cases were associated with virus‐contaminated sewage systems, which may contain SARS coronavirus in the wastewater and excreta. Considering that SARS‐CoV‐2 may enter wastewater through the excreta of infected individuals, it is necessary to implement appropriate measures to prevent potential risks.^[^ ^7^ ^]^ This revealed that the transmission capacity of SARS‐CoV‐2 in wastewater poses a potential threat to human health.

Over the years, multiple water purification techniques have been used to inactivate waterborne pathogens, including chlorine disinfection, ultraviolet (UV) irradiation, and ozone disinfection.^[^ ^17^ ^]^ However, these methods have disadvantages, such as low efficiency, high cost, and environmental pollution. As an advanced oxidation technology, atmospheric‐pressure plasma has the advantages of high efficiency and safety, and has attracted interest for biomedical sciences and wastewater purification.^[^ ^18^, ^19^, ^20^, ^21^, ^22^, ^23^, ^24^ ^]^ In particular, cold atmospheric plasma (CAP) generated by a pulse power supply provides a low temperature while maintaining high reactivity, and has made a significant contribution to coronavirus‐contaminated surface disinfection.^[^ ^25^, ^26^ ^]^ A series of oxidation reactions and physical effects occur during the CAP treatment process, resulting in the generation of a large number of reactive species that are responsible for virus disinfection.^[^ ^27^, ^28^, ^29^ ^]^

Although several studies have shown that CAP can inactivate viruses and bacteria in wastewater,^[^ ^30^ ^]^ the mechanism of plasma–water interaction is extremely complicated and unclear. Additionally, no research has been conducted on the use of CAP to treat wastewater containing SARS‐CoV‐2. In contrast to surface SARS‐CoV‐2 disinfection, water treatment involves gas‐phase electron and reactive species generation, water–liquid interface and liquid reactions, making the disinfection mechanism more complex. Based on the advantages of CAP, investigating its capability and mechanism of inactivation of SARS‐CoV‐2 in wastewater is a high priority. Of particular interest is the significant difference between this study and our previous report on surface disinfection of coronaviruses. In our previous report, an atmospheric pressure low‐temperature plasma jet device was employed to generate plasma containing a significant number of reactive species, which were transported directly to the treated surface in the form of a plume, leading to virus inactivation. A virus volume of 25–300 μL was used to perform a preliminary exploration of the surface inactivation capability and mechanism of CAP.^[^ ^26^ ^]^ In contrast, in this study, we developed a liquid‐phase corona plasma discharge device to investigate in depth the effectiveness and mechanism of CAP in inactivating coronaviruses in large volumes of water. This approach is motivated by the significant public health threat posed by the presence of SARS‐CoV‐2 in wastewater in various regions of the world. The corona discharge appears near a sharp electrode geometry and extends to the water surface under electric field forces. Reactive species are first generated above the water surface and spread into the water layer with the plasma propagation, resulting in virus inactivation within the water.

In this study, we examined the capability of CAP to inactivate SARS‐CoV‐2 in a large volume of water using SARS‐CoV‐2 transcription‐ and replication‐competent virus‐like particles (trVLP), pseudotyped SARS‐CoV‐2 variants and the SARS‐CoV‐2‐like coronavirus GX_P2V. In addition, CAP was evaluated for coronavirus inactivation using porcine epidemic diarrhoea virus (PEDV). Viral attachment assays, enzyme‐linked immunosorbent assays (ELISAs), western blotting, and viral RNA detection were performed to investigate the inactivation of coronaviruses by CAP. Importantly, our study provides a more efficient and safer option for wastewater disinfection during the COVID‐19 pandemic.

## 2 RESULTS

### 2.1 Cold atmospheric plasma effectively disinfected GX_P2V and SARS‐CoV‐2 virus‐like particles in a large volume of water

**FIGURE 1:**
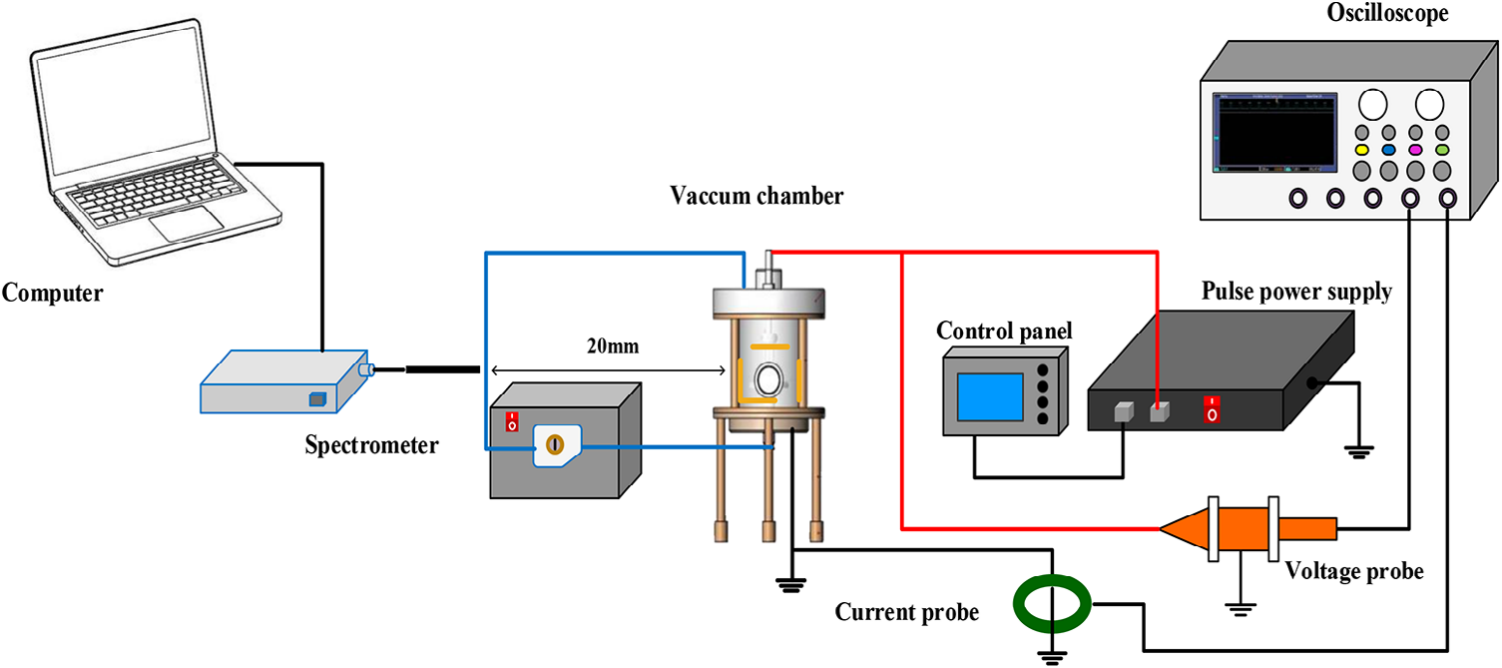
Schematic diagram of the cold atmospheric plasma device used for coronavirus inactivation.

For evaluating the potential of CAP to inactivate SARS‐CoV‐2 in a body of water, we designed a device to simulate the recycling of wastewater for laboratory use (Figure 1). The circulation unit serves to ensure that the liquid in the CAP device undergoes a more complete and uniform treatment with the virus solution. This is achieved by connecting two plastic pipes below the plasma unit, which carry the virus solution through a peristaltic pump and into the liquid inlet above the CAP device. The circulation pump is set to a flow rate of 71 mL min^‐1^, which allows the solution to pass through the pipes and the peristaltic pump, promoting circulation and ensuring a more homogeneous treatment of the liquid in the CAP device. Viral inactivation evaluation was performed using the pangolin coronavirus GX_P2V, which has been widely used as an alternative model for SARS‐CoV‐2 studies in our previous work. Simultaneously, the replication‐competent SARS‐CoV‐2 trVLP was used to verify the results of the GX_P2V model. The amino acid of the spike protein of GX_P2V has 92.2% homology with SARS‐CoV‐2 and is considered an excellent replacement model for SARS‐CoV‐2.^[^ ^31^ ^]^ The SARS‐CoV‐2 trVLP expresses green fluorescent protein (GFP), a reporter gene, to replace the viral N gene, which is essential for SARS‐CoV‐2 genome packaging and viral particle assembly. The trVLP completes its life cycle in Caco2‐N cells ectopically expressing the SARS‐CoV‐2 N protein, and can be manipulated in the BSL‐2 laboratory.^[^ ^32^ ^]^

**FIGURE 2:**
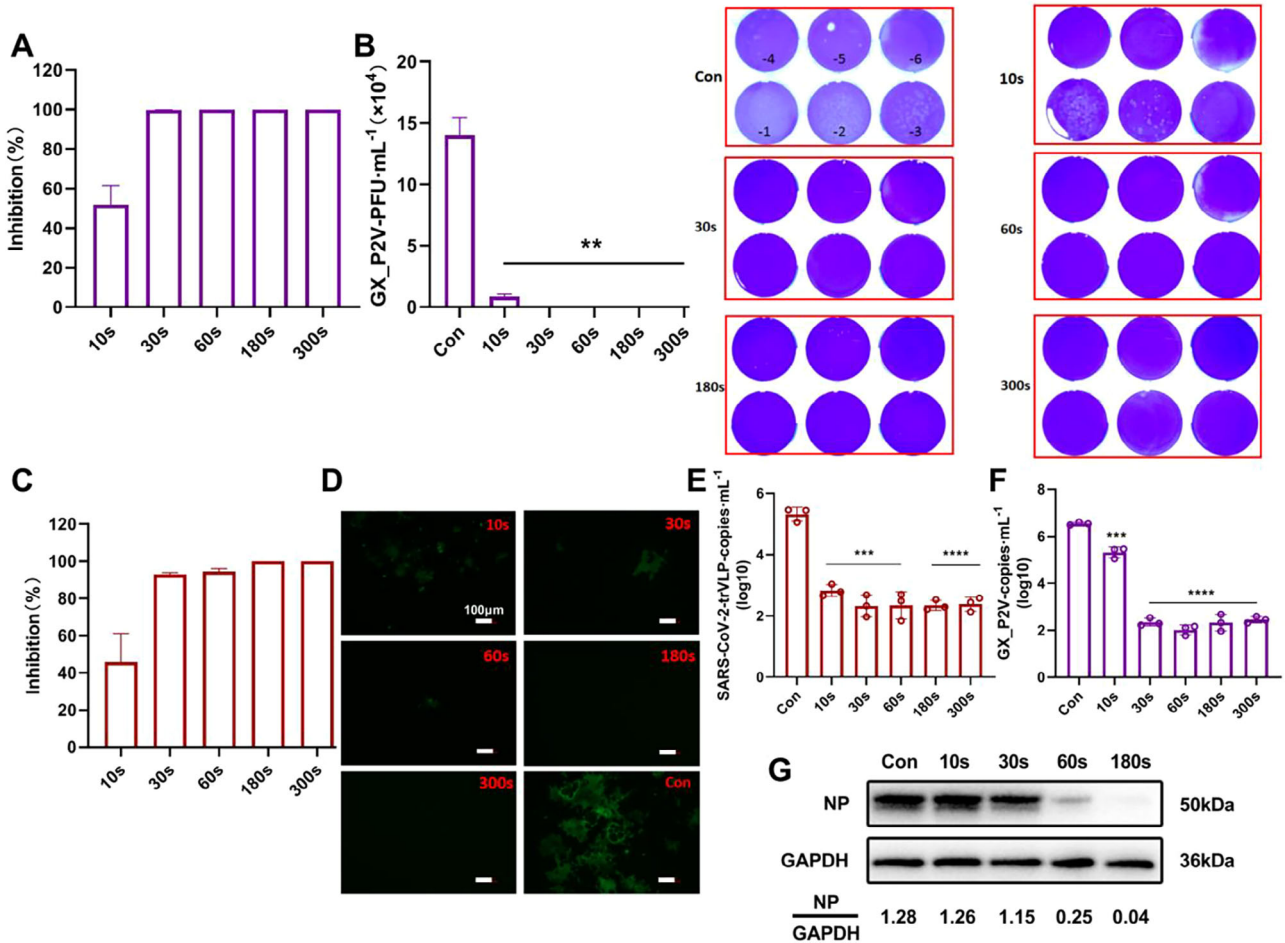
The effect of exposure to cold atmospheric plasma (CAP) on coronavirus GX_P2V and SARS‐CoV‐2 transcription‐ and replication‐competent virus‐like particle (trVLP) infection in a volume of water. (A, C) Inhibition (%) of GX_P2V and SARS‐CoV‐2 trVLP at various CAP exposure durations. (B) Plaques produced by GX_P2V at various CAP exposure durations and quantified. (D) Changes of in the green fluorescent protein fluorescence signal of SARS‐CoV‐2 trVLP after CAP treatment for varying lengths of time. (E–G) Effect of CAP on replication, assembly and ability to produce progeny coronaviruses.

We added 180 mL of water with GX_P2V (5 × 10^5^ PFU mL^‐1^) or SARS‐CoV‐2 trVLP (2.3 × 10^3^ TCID_50_ mL^‐1^) to the wastewater simulation device with the circulation system open. The infectivity of GX_P2V after CAP treatment was quantified using a TCID_50_ assay. As shown in Figure 2, GX_P2V infectivity decreased in a stepwise manner as CAP treatment time increased. After 30 s of CAP treatment, the inactivation rate reached 99%, and increased to over 99.9% after 60 s of treatment, indicating an almost complete elimination of the infectious virus. Further, the cytopathic effect of GX_P2V infection on Vero E6 cells was significantly reduced after 30 s of treatment and was hardly observed after 60 s of treatment, following the cellular morphology was similar to that of normal cells (Figure 2A and Figure S2). The plaque assay also confirmed the above results, with the observation of a drastic reduction in plaques formed by CAP‐treated GX_P2V cells, indicating that the infection ability of the virus was significantly abolished (Figure 2B). To further investigate the ability of CAP to inactivate SARS‐CoV‐2 in a large volume of water, we used SARS‐CoV‐2 trVLP to validate the above results. For SARS‐CoV‐2 trVLP inactivation, 30‐s CAP treatment inhibited viral infection by 93%, and the 180‐s treatment achieved over 99% inhibition, with undetectable GFP fluorescence signals in Caco2‐N cells (Figure 2C, D). We infected host cells with CAP‐treated samples and measured the viral copies in the supernatant after 48 h of incubation. The ability of SARS‐CoV‐2 trVLP and GX_P2V to produce progeny virus was severely impaired (Figure 2E, F), number of copies of virus decreased from 5.32 to 3.38 log10 copies mL^‐1^ (*p* < 0.0001), and from 6.54 to 2.45 log10 copies mL^‐1^ (*p* < 0.0001), respectively, indicating a significant reduction in the level of progeny production. Furthermore, GX_P2V treated with CAP for different durations infected host cells, and intracellular proteins were collected after 48 h of infection to detect the expression of N protein by western blotting. N protein expression significantly decreased in a treatment time‐dependent pattern (Figure 2G), which indicated that CAP affected the replication and assembly of the progeny virus.

These results show the effectiveness of CAP at inactivating SARS‐CoV‐2 in a large volume of water. Previous studies have shown the broad‐spectrum bacterial inactivation ability of CAP.^[^ ^33^, ^34^ ^]^ In this study, we aimed to identify the effectiveness of CAP in disinfecting coronaviruses in a large volume of water. Next, we investigated the inactivation of PEDV using CAP. PEDV inactivation was 36.3% and 99.9% after 30 and 180 s of CAP treatment, respectively (Figure S3). Given the morphological and structural identity of coronaviruses, CAP can inactivate coronaviruses in a large volume of water.

### 2.2 Cold atmospheric plasma reduced attachment of pseudotyped SARS‐CoV‐2 variants and coronaviruses to cells

**FIGURE 3:**
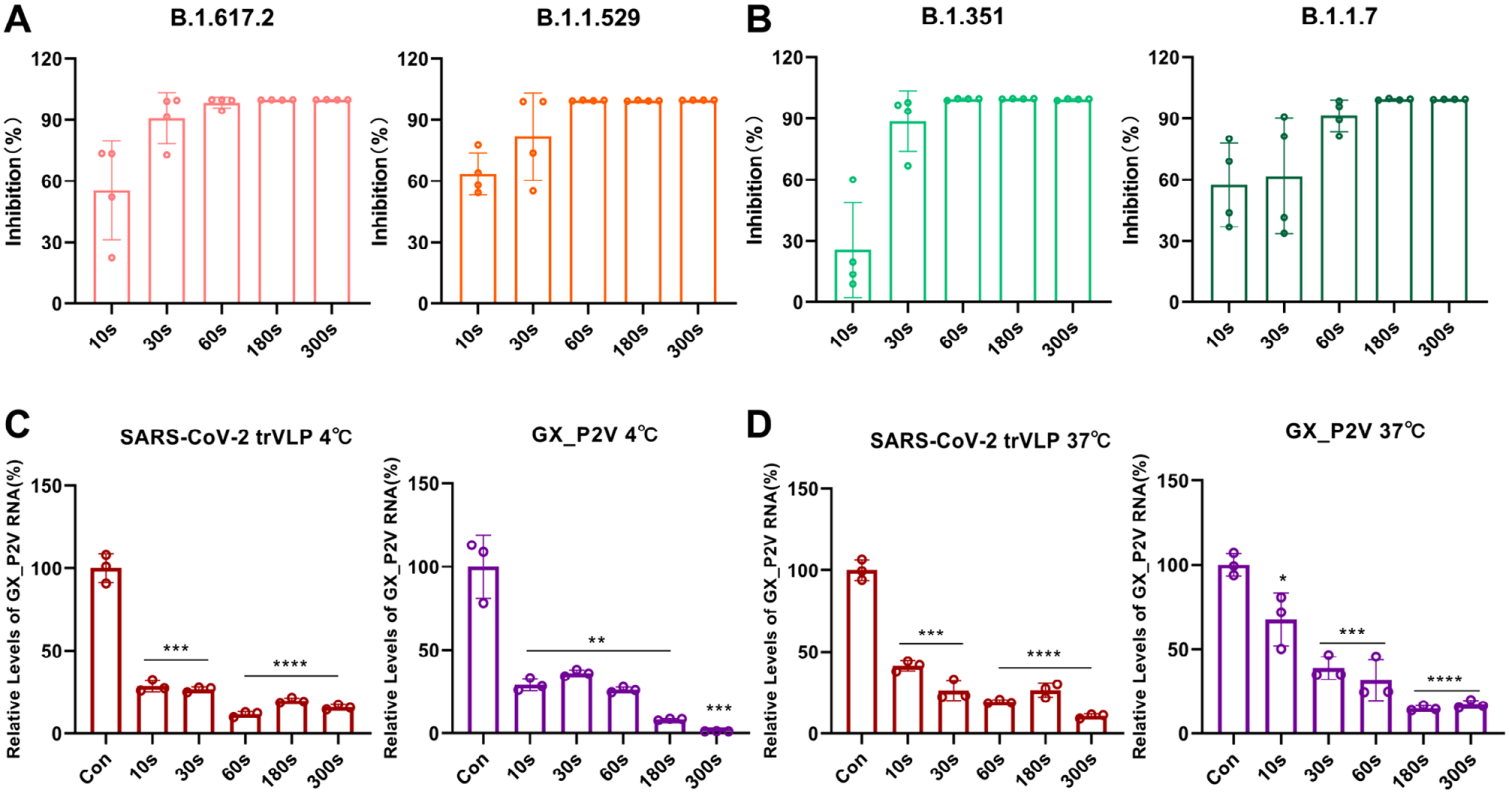
Inactivation of SARS‐CoV‐2 pseudovirus variants in a large volume of water by cold atmospheric plasma (CAP). (A) Inactivation of Delta‐ and Omicron‐pseudovirus by CAP. (B) Inactivation of Beta‐ and Alpha‐pseudovirus by CAP. (C) Ability of CAP‐treated coronaviruses to bind to host cells. (D) Ability of CAP‐treated coronaviruses to enter cells.

To further investigate the inactivation ability of CAP on SARS‐CoV‐2 in a large volume of water, we tested the effect of CAP on the pseudotyped SARS‐CoV‐2 variants. In this study, 30 mL of SARS‐CoV‐2 pseudovirus and 150 mL of water (with 0.22 μm filtration) were uniformly mixed and added to the wastewater simulation device. The RLU in the CAP treatment group was significantly lower than that in the untreated group, and the reduction was time‐dependent. Notably, after 300 s of CAP treatment, the RLU was almost identical to that of the blank control, with RLU of B.1.617.2 (Delta) and B.1.1.529 (Omicron) decreasing from 1,670 and 60,228 to 4.8 and 495, and inhibition rates calculated as 99.73% and 99.58%, respectively (Figure 3A). The RLU of B.1.351 (Beta) and B.1.1.7 (Alpha) decreased from 100,715 and 85,145 to 747 and 611, respectively, with inhibition rates of 99.26% and 99.28%, respectively (Figure 3B). We confirmed that CAP can inactivate SARS‐CoV‐2 in a large volume of water. Based on the structure of the pseudovirus, we speculated that inactivation of the spike protein might be one of the disinfection mechanisms of CAP on SARS‐CoV‐2. The spike is important for coronavirus recognition and infection of host cells. This process can be explained by the attachment and internalisation assays of live viruses. SARS‐CoV‐2 trVLPs and GX_P2V treated with CAP were incubated with Caco2‐N and Vero E6 cells at 4°C (for viral attachment only) and 37°C (for both viral attachment and internalisation), respectively. The level of virus copies on the cell membrane was detected to reflect the amount of virus that was attached and internalised. The attachment of SARS‐CoV‐2 trVLP and GX_P2V after 300 s of treatment decreased by 84% (*p* < 0.0001) and 98% (*p* = 0.0008), respectively (Figure 3C), and viral internalisation decreased by approximately 90% (*p* < 0.0001) and 82% (*p* < 0.0001), respectively (Figure 3D). The results indicated that CAP significantly reduced the ability of coronaviruses in water to attach and enter host cells by inactivating the spike protein. In our previous surface disinfection studies of CAP, TEM electron microscopy images were captured of live viral particles treated with CAP for different durations. The results revealed that as the treatment time increased, the morphology of the viruses exhibited shrinkage and collapse, indicating an impact on viral attachment to host cells.^[^ ^26^ ^]^ After treatment for 180 s by CAP, distinct viral morphological changes were observed, and most of the particles exhibited shrunken and irregular spherical structures. These results indicate that CAP destroyed the viral outer membrane structure, in which the S protein was anchored.

### 2.3 Cold atmospheric plasma disrupted the coronavirus spike protein

**FIGURE 4:**
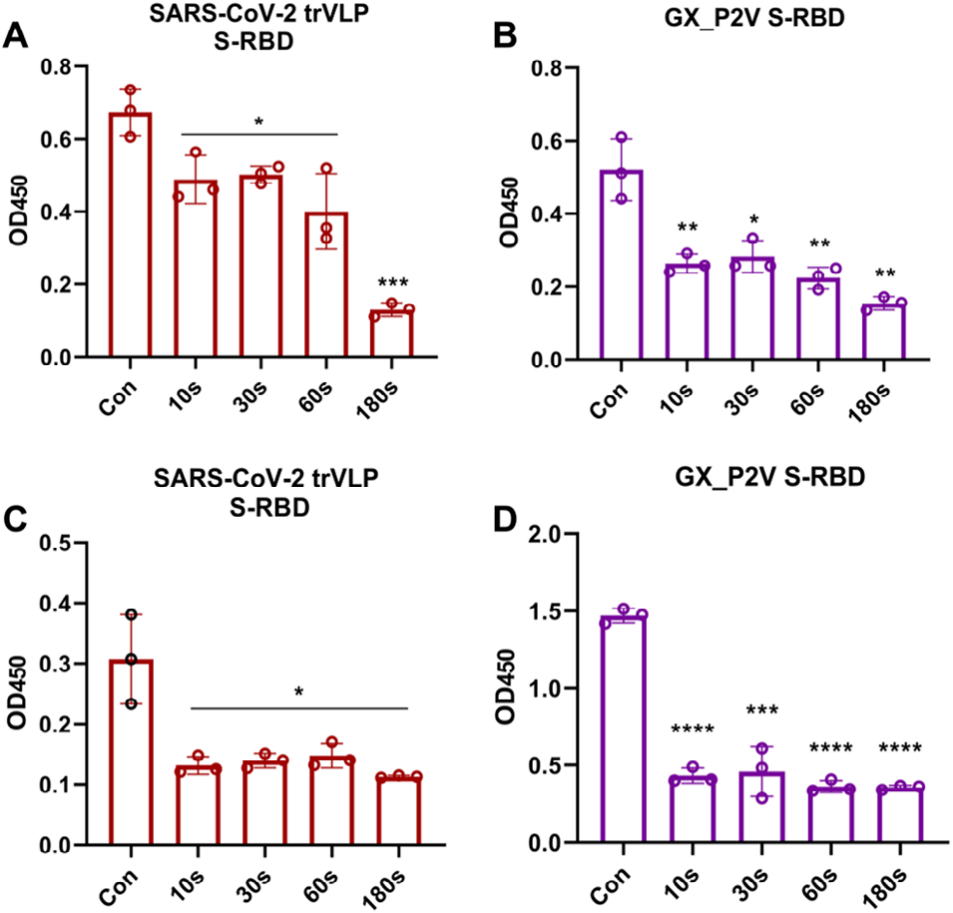
Activity of coronaviruses spike after cold atmospheric plasma (CAP) treatment. (A, B) Detection of receptor‐binding domain (RBD) activity of coronaviruses treated with CAP using an enzyme‐linked immunosorbent assay. (C, D) Detection of RBD activity in the supernatant after infection of cells with CAP‐treated coronaviruses. The columns on the left of each figure are the control sample. The other samples have been exposed to CAP for periods ranging from 10 s (second column) to 180 s (right column).

Next, we investigated the effect of CAP on spike protein using ELISA. The receptor‐binding domain (RBD) of the spike protein, located in the S1 subunit, is responsible for the recognition of the receptor on the host cell surface. A total of 180 mL of the virus suspension (SARS‐CoV‐2 trVLP or GX_P2V) was added to the wastewater simulation device and subjected to CAP disinfection. The samples were collected at 10, 30, 60, and 180 s of treatment to detect the ACE2 binding ability. The activity of SARS‐CoV‐2 trVLP RBD decreased moderately after 60 s of treatment (*p* = 0.018, Figure 4A), and decreased significantly after 180 s of treatment (*p* = 0.0002, Figure 4A). The activity of GX_P2V RBD decreased significantly after 60 s of treatment (*p* = 0.0046, Figure 4B). For further verification, we infected Caco2‐N and Vero E6 cells with CAP‐treated water samples for 2 h, and then replaced them with fresh medium and continued the culture for 48 h. The disruption of the RBD affected viral entry and replication, suppressing the production of progeny virions (Figure 4C, D).

### 2.4 Cold atmospheric plasma damaged coronavirus RNA

**FIGURE 5:**
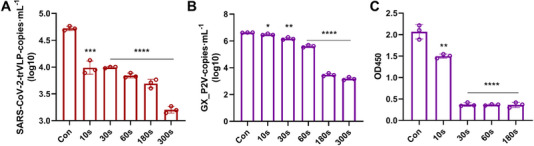
Effect of cold atmospheric plasma (CAP) on coronavirus replication. (A, B) Effect of CAP on coronaviruses copy numbers in a large volume of water. (C) Effect of CAP on coronavirus nucleocapsid (N) protein.

Given that viruses in a large volume of water retained a weak ability to enter cells after CAP treatment, we hypothesised that the mechanism of CAP may not only damage the viral spike on the surface. Thus, the effect of CAP on coronavirus RNA was further investigated. Samples were collected at various time points (10, 30, 60, 180, and 300 s) after CAP treatment, followed by RNA extraction for reverse transcription. RNA copy numbers were determined using RT‐qPCR. The RNA copy numbers of both SARS‐CoV‐2 trVLP and GX_P2V decreased significantly after CAP treatment (Figure 5A, B). The RNA copies of SARS‐CoV‐2 trVLP decreased from 4.72 to 3.20 log10 copies mL^‐1^ (*p* < 0.0001) after 300 s treatment. Similarly, the RNA copies of GX_P2V decreased from 6.62 to 3.18 log10 copies mL^‐1^ (*p* < 0.0001). These results confirmed that CAP treatment can disrupt the RNA structure of coronaviruses in water samples.

As viral N protein distributed on the surface of viral RNA, it can form Ribonucleoprotein (RNP) complexes with viral RNA to protect the viral genome. Hence, we also investigated the effect of CAP on the viral N protein to further explain its powerful ability to inactivate coronaviruses in a large volume of water. The SARS‐CoV‐2 N protein is a structural protein that combines and encapsidates the viral genome and is abundantly expressed in infected cells. It participates in the assembly and replication of coronavirus.^[^ ^35^ ^]^ We hope to achieve a comprehensive understanding of the inactivation mechanism of CAP on coronavirus by studying the N protein. The activity of GX_P2V N protein was determined using ELISA. The OD_450_ significantly decreased after 30 s of treatment compared with that of the control group (*p* < 0.0001, Figure 5C), indicating that the functional GX_P2V N protein was reduced after CAP treatment.

### 2.5 Cold atmospheric plasma disinfection had negligible cytotoxicity

**FIGURE 6:**
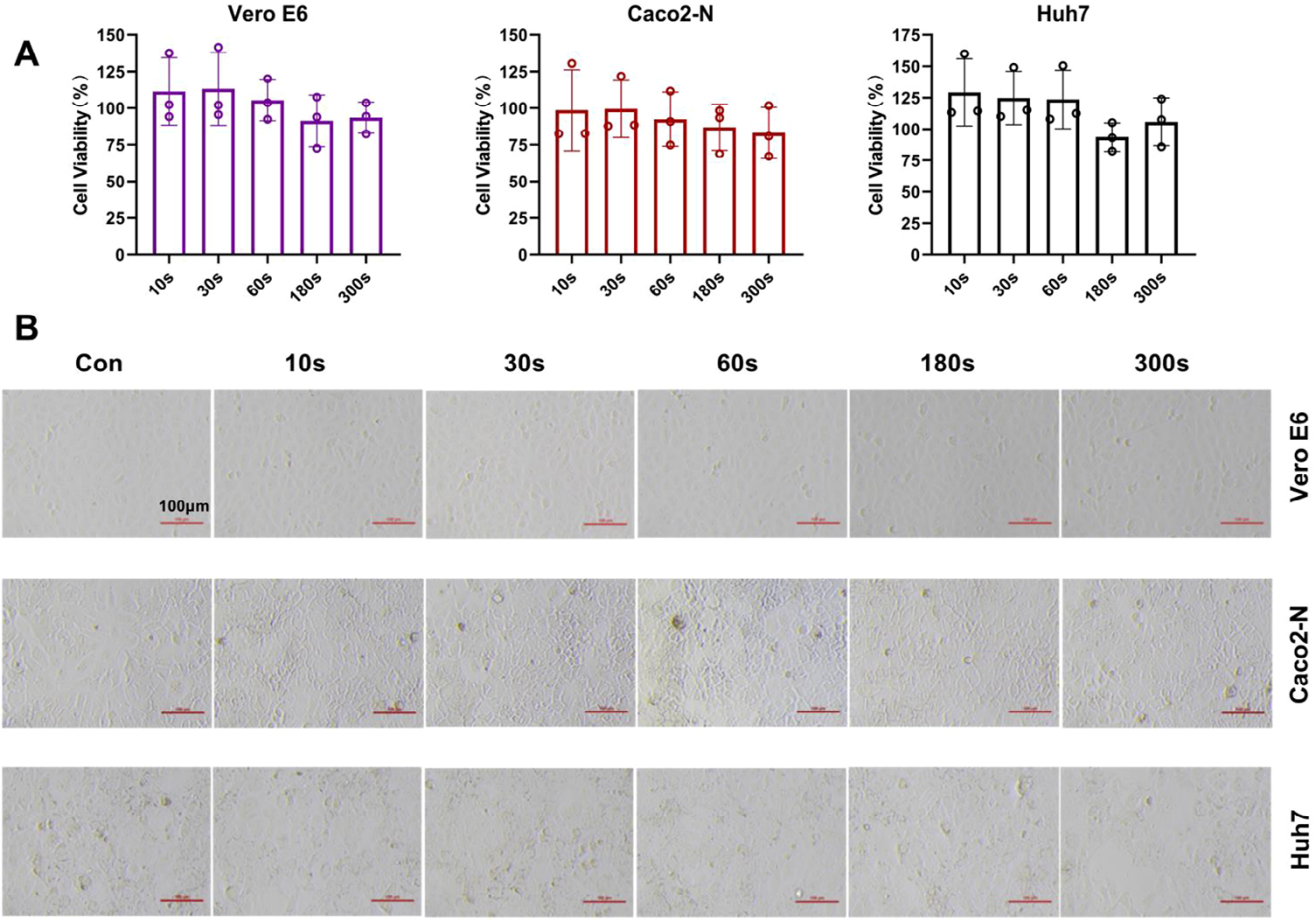
Effect of cold atmospheric plasma (CAP) on cell viability. (A) Cell viability after varying periods of CAP treatment, ranging from 10 to 300 s. Left: Vero E6; middle: Caco2‐N; Bottom: Huh7. (B) Comparison of cell morphology after culturing cells with CAP‐treated medium. Top row: Vero E6; middle row: Caco2‐N; Bottom row Huh7.

As CAP shows an excellent disinfection effect on coronaviruses, the biosecurity of CAP treatment should be confirmed to facilitate practical application. Vero E6, Caco2‐N, and Huh7 cells were cultured in a culture medium which was pre‐exposed to CAP for various durations. Cell viability was examined after 48 h of incubation using the CellTiter‐Blue method. Cell viability was unaffected under all tested conditions, even if cultured in the medium after 300 s of CAP treatment (Figure 6A). Moreover, the cell morphology of the treatment groups showed no obvious differences from the control group under a microscope (Figure 6B). Using three mammalian cell lines, we demonstrated that CAP treatment is safe for cells and even promoted cell proliferation when the culture medium was pre‐treated with CAP for less than 60 s.

### 2.6 Reactive free radicals in plasma–liquid systems

**FIGURE 7:**
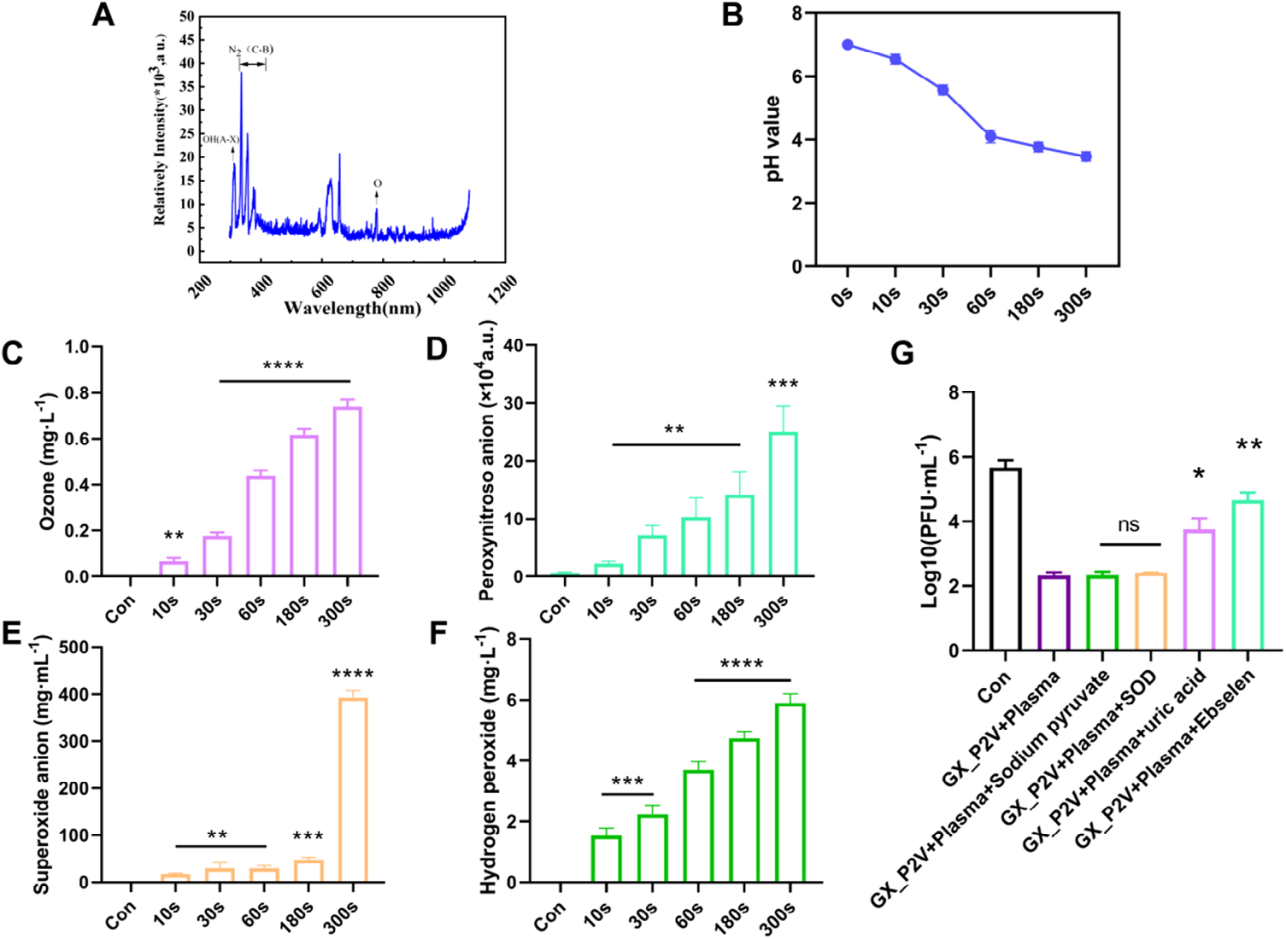
Active substance measurement and quenching experiments. (A) Optical emission spectra of pulsed coronavirus plasma. (B) Temperature and pH variations in the water after varying durations of cold atmospheric plasma (CAP) exposure. (C) Ozone (O_3_) levels in the water after varying durations of CAP exposure. (D) Peroxynitrite (ONOO^−^) levels in the water after varying durations of CAP exposure. (E) Superoxide anion (O_2_^−^) levels in the water after varying durations of CAP exposure. (F) Hydrogen peroxide (H_2_O_2_) levels in the water after varying durations of CAP exposure. (G) The titres of GX_P2V premixed with quencher after 60 s of treatment with CAP were measured. (**p < 0.01).

To investigate the reactive species at the plasma–liquid interface, emission spectroscopy was used to characterize the reactive species produced by the reaction (Figure 7A). Reactive nitrogen species (RNS) N_2_ (N_2_ second‐positive system) and ionised nitrogen molecules N_2_^+^ (N_2_^+^ first‐negative system) were detected in the 300−420 nm and 400−500 nm ranges, respectively. Furthermore, an OH (A‐X) band was observed at 306−312 nm, and emission peaks at 426 and 777.3 nm were associated with radiogenic oxygen. The emissions above 600 nm may be the second order from OH and Nitrogen bands. These peaks indicate the production of reactive oxygen species (ROS) and RNS during the plasma–water reaction. RONS are thought to be the first components of plasma–water interactions and directly influence the formation of chemical species in the liquid phase.^[^ ^36^ ^]^ The chemical reaction between gaseous RONS and H_2_O determines the concentration of water‐based active species, which in turn determines the efficiency of virus disinfection. Based on this reaction, long‐lived species such as HNO_3_, HNO_2_, and H_2_O_2_ were generated, which induced pH changes. The pH value was reduced from 7 to 3.5, after 300 s of plasma treatment (Figure 7B), indicating that plasma treatment causes water acidification. After the plasma discharge reaction, the low pH can act as a catalyst, promoting the antimicrobial effect of reactive oxygen species.^[^ ^37^ ^]^

RONS scavengers were added to the water sample to determine the role of long‐ and short‐lived substances generated by plasma in virus inactivation. Prior to CAP exposure, uric acid (100 m), sodium pyruvate (10 mm), superoxide dismutase (SOD) (500 U mL^‐1^), and ebselen (1 mm), which are scavengers of O_3_, H_2_O_2_, O_2_^−^, and ONOO^−^, were premixed with GX_P2V. The GX_P2V disinfection effect of CAP was offset by the addition of these scavengers (Figure 7G). Ebselen effectively eliminated the inactivation effect of CAP, with infectious virus production increased by 2.34 orders of magnitude, indicating that ONOO^−^ played a critical role in the CAP disinfection process. Uric acid reduced the disinfection efficacy of CAP by 1.425 orders of magnitude. Uric acid preferentially quenched OH (reaction constant: 7.2 × 10^6^) and O_3_ (reaction constant: 1.4 × 10^6^) and partially quenched ONOO^−^. Conversely, SOD and sodium pyruvate had negligible effects on CAP disinfection, showing that O_2_^−^ and H_2_O_2_ produced using CAP made little contribution to the virus inactivation. We measured the O_3_ content in the plasma–liquid system to confirm O_3_ generation using the CAP device. The O_3_ content increased as CAP treatment time increased, and 0.6 ppm of O_3_ was detected after 300 s of CAP treatment (Figure 7C). We hypothesised that ONOO^−^ plays a vital role in virus inactivation and assessed the ONOO^−^ level to confirm its existence in the plasma–liquid system. The concentration of ONOO^−^ was positively associated with the CAP treatment time (Figure 7D). We conducted measurements on the concentrations of O_2_^−^ and H_2_O_2_ within the plasma–liquid system. Despite their relatively marginal influence on the process of virus inactivation, we were able to detect the presence of both substances. The concentration of both substances tended to increase with the CAP treatment time. Specifically, the O_2_^−^ concentration reached 392.56 mg mL^‐1^ after 300 s of processing (Figure 7E), whereas the H_2_O_2_ concentration reached 5.9 ppm after 300 s of processing (Figure 7F).

**FIGURE 8:**
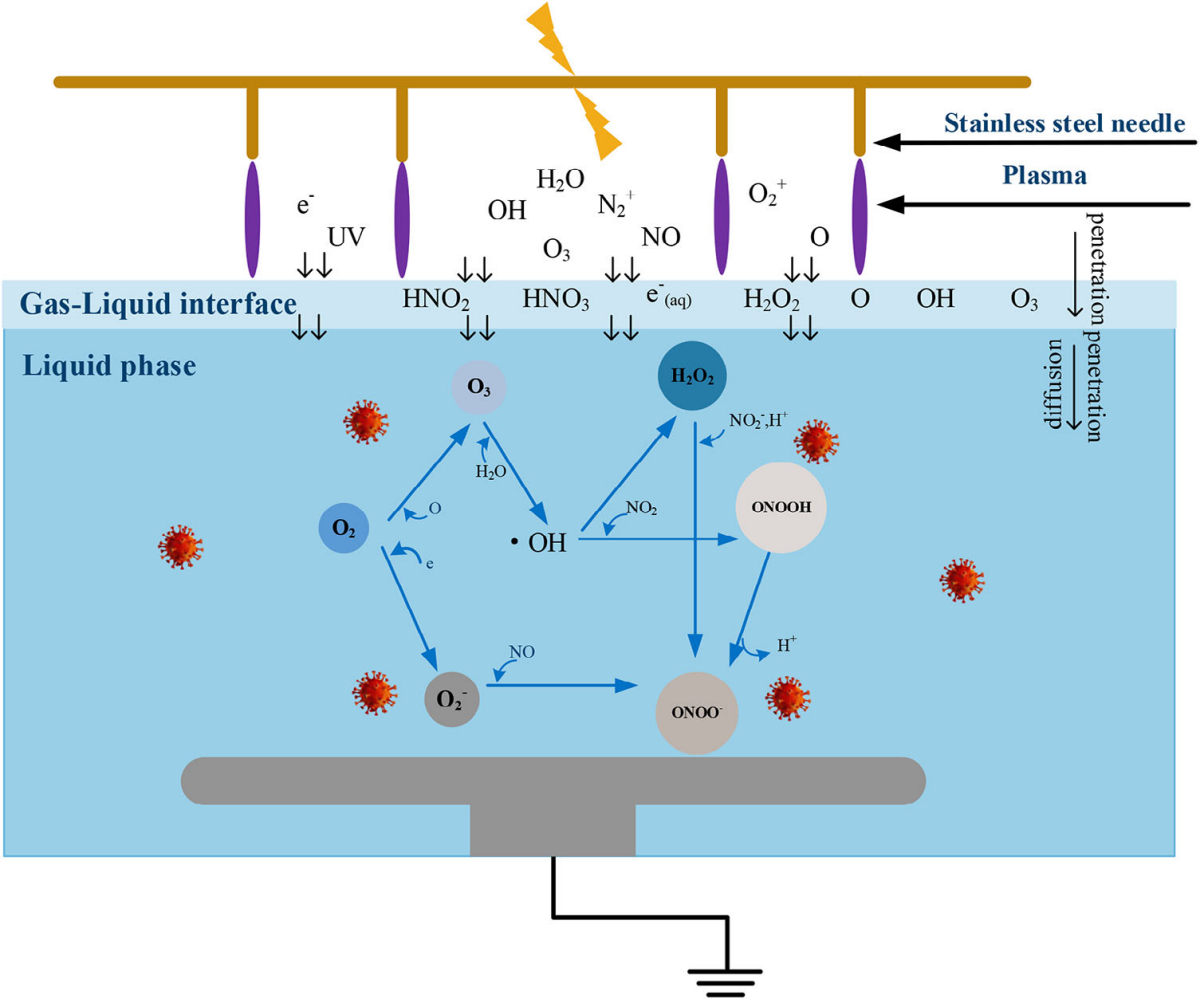
Schematic diagram of formation of reactive species in cold atmospheric plasma.

The plasma–liquid system undergoes a series of reactions in the presence of RONS to produce a large number of reactive substances, can be divided into two categories: short‐lived substances, including O_2_^−^, OH, and ONOO^−^, and long‐lived substances, including O_3_, NO_3_^−^, NO_2_^−^, and H_2_O_2_, which exist for a few days. Although these reactive substances have distinct lifetimes, they are able to transform through a variety of processes and share the same plasma treatment process. During the course of the treatment, plasma constituents such electrons, photons, radicals, and gas‐phase ROS and RNS reach the interface, diffusing and activating chemical reactions with the production of liquid–phase chemical species. These species can then diffuse away from the interface and penetrate the liquid phase, where further chemical processes take place. For example, O_3_ is formed by the collision between O_2_, oxygen atoms, and the third body M (O, O_2_, or O_3_) via reaction (1).^[^ ^38^ ^]^ O_3_ reacts with H_2_O to produce OH and O_2_ via reaction (2). High‐energy electrons collide with O_2_ to produce O_2_^−^ via reaction (3).^[^ ^39^ ^]^ OH is considered an intermediate substance in the plasma reaction, and can self‐aggregate to form H_2_O_2_, and combine with NO_2_ to form ONOOH via reactions (4, 5).^[^ ^40^ ^]^ ONOO^−^ can be formed via the reaction of NO_2_^−^ and O_2_^−^ as well as the reaction of NO_2_^−^ with H_2_O_2_ under acidic conditions and the decomposition of ONOOH via reaction ((6), (7), (8))^[^ ^33^, ^34^ ^]^ (Figure 8).

(1) $$O+O_{2}+M\to O_{3}^{*}+M\to O_{3}+M$$

(2) $$O_{3}+H_{2}O\to 2\;\cdot \mathrm{OH}+O_{2}$$

(3) $$O_{2}+e\to {O_{2}}^{-}$$

(4) $$\cdot \mathrm{OH}+\cdot \mathrm{OH}\to H_{2}O_{2}$$

(5) $$NO_{2}+\cdot \mathrm{OH}\to \mathrm{ONOOH}$$

(6) $$\mathrm{ONOOH}\to \mathrm{ONO}O^{-}+H^{+}$$

(7) $$\mathrm{NO}+{O_{2}}^{-}\to \mathrm{ONO}O^{-}$$

(8) $$N{O_{2}}^{-}+H_{2}O_{2}+H^{+}\to \mathrm{ONOOH}+H_{2}O$$

## 3 DISCUSSION

The COVID‐19 pandemic is ongoing, and water contaminated with SARS‐CoV‐2 may be a serious threat to public health. It is crucial to establish an efficient, safe, and environmentally friendly disinfection strategy for wastewater to prevent secondary epidemics caused by virus transmission through water. In this study, we developed a high‐voltage coronavirus pulse discharge device based on plasma technology that employs high‐voltage pulses to generate highly reactive antiviral factors, including RNS and ROS. Our CAP‐discharge device showed an efficient effect on reducing coronavirus loads and infectivity in a large volume of water. This is consistent with the antiviral performance of other devices with different types of plasma sources. Most antiviral studies using plasma have focused on virus disinfection on object surfaces or in a gaseous environment.^[^ ^41^, ^42^, ^43^ ^]^ However, to our knowledge, research on the use of CAP to inactivate SARS‐CoV‐2 in a large volume of water has not been reported previously. In this study, we investigated for the first time the efficacy of CAP on inactivating coronaviruses in a large volume of water. CAP inactivated most of the viruses in water samples in a short time. Infectious GX_P2V production decreased by >99.9% after 60 s of CAP exposure, and SARS‐CoV‐2 trVLP production decreased by >99.0% after 180 s of CAP exposure. Unlike GX_P2V, SARS‐CoV‐2 trVLP replicates by utilizing the host cell‐expressed N protein. Differences in viral structure, composition, replication cycle, and host cells may contribute to variations in the efficacy of CAP in inactivating these viruses. However, the trend of inactivation on coronaviruses remains unchanged. For the pseudotyped SARS‐CoV‐2 variants, the inhibition rate was > 99.2% after 300 s of treatment. CAP treatment significantly inhibited PEDV, indicating that CAP had a broad‐spectrum ability to inactivate coronaviruses. In contrast to Guo et al., who employed plasma‐activated water (PAW) to inactivate wild‐type pseudovirus of SARS‐CoV‐2,^[^ ^27^ ^]^ our study not only utilized multiple mutant strains of SARS‐CoV‐2 pseudovirus, and more importantly, systematically investigated the efficacy of CAP‐mediated virus inactivation in a large volume of water on coronavirus GX_P2V and SARS‐CoV‐2 trVLP models, both of which possess a complete life cycle.

The disinfection ability of CAP is attributable to interaction of oxidative reactive species and viral particles. The gaseous RONS (NO_x_, OH, H_2_O_2_, HNO_2_, HNO_3_, O_3_) in CAP penetrate or dissolve into liquids and initiate chemical reactions that produce aqueous RONS. The common aqueous species in plasma‐activated water are long‐lived species, including H_2_O_2_, NO_2_^−^, and NO_3_^−^, and transient species, including ONOO^−^/ONOOH, O_2_^−^, OH, NO, and ·NO_2_.^[^ ^44^ ^]^ To verify the contribution of the generated RONS to virus inactivation, we silenced reactive ions by adding specific scavengers. Ebselen, a specific ONOO^−^ scavenger,^[^ ^45^, ^46^ ^]^ reduced the CAP inactivation ability by 2.34 orders of magnitude, demonstrating the vital function of ONOO^−^ in coronavirus disinfection. ONOO^−^ is a physiologically relevant nitrogen species that is produced in the human body through biochemical reactions.^[^ ^47^ ^]^ During the pulsed coronavirus discharge process, ONOO*^−^* is formed through many reactions (reactions (5) to (8)). The effect of O_3_ is secondary to that of ONOO^−^. We identified the favourable antiviral ability of O_3_, with the quenching of O_3_ decreasing the disinfection ability of CAP by 1.425 orders of magnitude. Although the antiviral ability of O_3_ in most reported CAP disinfection strategies is limited,^[^ ^48^ ^]^ few studies have identified O_3_ as an important substance for FCV inactivation by CAP.^[^ ^49^, ^50^, ^51^ ^]^. The concentration of O_3_ produced was 0.6 mg L^‐1^ at 300 s treatment. Martins et al. showed that 0.6 ppm (0.6 mg L^‐1^) ozonated water reduced the virus titre by two orders of magnitude after a 1 min treatment.^[^ ^52^ ^]^ For enveloped viruses, O_3_ oxidises the viral envelope by modifying or disrupting its envelop structure. The absence of H_2_O_2_ and O_2_^−^ had a marginal effect on CAP disinfection, indicating that H_2_O_2_ and O_2_^−^ played marginal roles in the disinfection process. H_2_O_2_ is considered a weak oxidant; therefore, H_2_O_2_ may not cause significant damage to virus particles at the experimental concentrations that we used.

Two modes may explain the coronavirus inactivation ability of CAP. (i) Reactive species destroy viral spike, thereby reducing coronavirus infectivity. The ability of coronaviruses to attach and internalise, mediated by the spike RBD, was significantly impaired after CAP treatment, indicating that active substances weakened the binding ability of the spike to cellular receptors, thereby inhibiting coronavirus infection. The mechanism of reactive oxidative species was comprehensively analysed in our previous study of CAP surface disinfection. Cysteine, tryptophan, tyrosine, and methionine distributed on the surface of SARS‐CoV‐2 and GX_P2V spike proteins are susceptible to oxidation by active substances released from CAP.^[^ ^26^ ^]^ Tyrosine and tryptophan are highly sensitive to ONOO^−^ and are modified by oxidation.^[^ ^27^ ^]^ RONS produced by plasma leads to carbonylation of the spike, which impairs the binding of the spike to the human ACE2 receptor. This may depend on the specific virus and plasma source type employed. (ii) Reactive species destroy the coronavirus genome. The copy number of viral RNA in a large volume of water decreased significantly after CAP treatment (Figure 5), indicating that CAP damaged the coronavirus genome. Song et al.^[^ ^28^ ^]^ found that reactive species reacted with the phosphoprotein and polymerase complex of spring viremia of carp virus in a large volume of water and prevented infection by damaging the genome. The genome of the waterborne pepper mild mottle virus was also damaged by CAP.^[^ ^53^ ^]^ Although previous findings have confirmed our speculation on the mechanisms of CAP inactivation of coronaviruses in water, the effect of CAP on the SARS‐CoV‐2 genome in a large volume of water requires further research. We explained the inactivation mechanism of CAP on SARS‐CoV‐2 in a large volume of water and found that CAP directly destroyed the N protein on the surface of viral RNA, providing a new insight into CAP inactivation of coronaviruses. In addition, the pH of the water sample decreased from 7.0 to 3.46 after CAP treatment for 300 s, which might have affected the survival of the virus^[^ ^54^ ^]^.

The safety of CAP in humans is a natural advantage of its use as a disinfectant. CAP exhibited negligible cytotoxicity to cells in vitro and in vivo and even tended to promote cell growth (Figure 6). Previous studies have reported that the reactive oxygen and nitrogen species (RONS) generated by CAP can serve as signalling molecules both intracellularly and extracellularly, directly or indirectly modulating the signalling pathways associated with cell proliferation. These bioactive species can affect cellular metabolism, proliferation, and differentiation, thereby facilitating the proliferation of multiple types of cells. Additionally, CAP treatment can induce the release of bioactive substances, such as cytokines, growth factors, and extracellular matrix proteins, which can promote cell proliferation and differentiation through their inherent biological activities and intercellular interactions.^[^ ^55^, ^56^ ^]^

The utilization of CAP on inactivating viruses in water demonstrates a rapid and highly efficient inactivation capability, enabling the substantial reduction of viral load within a short time, thereby mitigating the risk of infection. In particular, compared with conventional water treatment methods, plasma technology eliminates the need for chemical agents, thereby avoiding potential secondary contamination of water quality. However, the practical application of CAP faces certain challenges. Parameters such as processing capacity and flow rate of CAP may impact the disinfection efficacy, it is necessary to optimize various treatment conditions to enhance overall processing efficiency. Meanwhile, the operational and maintenance costs associated with CAP equipment should be taken into consideration during implementation. We simulated the large volume water circulation system in the laboratory during the study. Under realistic conditions, the wastewater composition may include organic substances, inorganic compounds, suspended solids, microorganisms etc. These constituents can potentially impact the disinfection efficacy of CAP.^[^ ^25^, ^57^ ^]^ For instance, suspended solids and organic substances may adsorb onto the surface of plasma‐producing instrument to reduce the quantity of plasma production or hinder its contact with pathogens. Microorganisms and organic substances can also influence the quantity and types of RONS generated by CAP, thereby affecting its disinfection performance.^[^ ^58^ ^]^ Hence, exploring the effects of CAP in the complex water matrices is the focus of our further studies. Additionally, constrained by research conditions, conducting investigations of CAP in large‐scale natural aquatic environments is challenging, as the widespread dispersion of infectious coronavirus particles could result in incalculable harm to the natural ecosystem. Therefore, within the laboratory setting, we made every effort to utilize larger volumes for validation. Our findings provide valuable indications for the inactivation efficacy of CAP in real‐world aquatic environments.

## 4 CONCLUSIONS

In summary, to investigate the capacity and mechanism of CAP in inactivating SARS‐CoV‐2 in wastewater, we developed a wastewater simulation device. We found that CAP efficiently inactivated SARS‐CoV‐2 trVLP, coronavirus GX_P2V, and pseudotyped SARS‐CoV‐2 variants within 180 s. CAP also had an effect on PEDV inactivation. Further mechanistic investigations showed that reactive species produced by CAP, such as ONOO^−^ and O_3_, disrupted coronavirus spike protein and the replication process, which are crucial to the inactivation of the virus. Finally, we showed that CAP inactivation did not have a negative effect on cell viability, demonstrating that CAP is an efficient and safe wastewater disinfection strategy. Overall, we have developed a broad‐spectrum, efficient, and safe strategy for the inactivation of coronaviruses in a large volume of water. Considering the widespread distribution of SARS‐CoV‐2, and possibility of emerging coronaviruses in wastewater in the future, we believe that the powerful disinfection capabilities of CAP may contribute to the prevention of waterborne SARS‐CoV‐2 spread. This work provides a promising water disinfection strategy and can be considered to combat water pollution by SARS‐CoV‐2 and other coronaviruses that might emerge in the future.

## 5 MATERIALS AND METHODS

### 5.1 Cold atmospheric plasma synthesis and characterization

Figure 1 shows a schematic diagram of the experimental setup. The CAP device was composed of seven needles and a lower stainless‐steel plate as high‐voltage and ground electrodes, respectively. Seven needles with a diameter of 3 mm and a length of 25 mm were evenly located in the centre of the upper stainless‐steel plate. Before treatment, 180 mL of a mixture of water and virus was poured into the coronavirus discharge reactor. The distance between the liquid surface and ground electrode was maintained at 10 mm, and the distance between the high‐voltage electrode and liquid surface was maintained at 5 mm. A peristaltic pump was used to circulate the liquid throughout the chamber to ensure adequate wastewater treatment. The pump circulation rate was 71 mL min^‐1^. The coronavirus discharge reactor was powered using a pulse power supply (Xi'an Smart Maple Electronic Technology, HVP‐22P, Xi'an, China). The voltage and current characteristics were monitored using a high‐voltage probe (Tektronix, P6015A, 1000:1, OR, USA) and a current probe (Pearson, Model 4100, 1 V/A), respectively, using a digital oscilloscope (Lecory WR204XI, NYC, USA). To obtain stable discharge, the applied voltage was maintained at 7 kV. A current peak was observed when the applied voltage reached its maximum value, confirming a strong discharge (Figure S1A). The average discharge power was then calculated by integrating the voltage and current during one discharge period, as described in our previous study.^[^ ^26^ ^]^ The average and instantaneous powers were calculated as 58.9 W and 4 kW, respectively (Figure S1B). The fibre optic cable was placed directly ahead of the discharge chamber at a spacing of 20 mm to capture the light emitted from the plasma plume to the spectrometer (Fuxiang, FX2000, Shanghai, China).

### 5.2 Virus and cell lines

Vero E6, Huh‐7, BHK‐21, and Caco2‐N cells were maintained in Dulbecco's modified Eagle's medium (HyClone, USA) supplemented with 10% foetal bovine serum (Gibco, USA) and 1% Antibiotic‐Antimycotic (Gibco, USA). The SARS‐CoV‐2‐related pangolin coronavirus GX_P2V (accession no. MT072864.1), isolated from smuggled dead Sunda pangolin (*Manis javanica*), was maintained and amplified in Vero E6 cells. SARS‐CoV‐2 trVLP‐expressing GFP, replacing the viral nucleocapsid gene, was proliferated in Caco2‐N cells.^[^ ^32^ ^]^ All the cells and viruses were cultured at 37°C in a 5% CO_2_ incubator.

### 5.3 Inactivation assays

The ability of CAP to inactivate coronaviruses in sewage was determined using the median tissue culture infectious dose (TCID_50_) and a plaque assay.^[^ ^26^ ^]^ A total of 180 mL of the GX_P2V suspension at a titre of 10^6^ TCID_50_ mL^‐1^ was subjected to a coronavirus discharge reactor. The movement of sewage was simulated using a circulation system (Figure 1). Viral samples were collected at different time points. For the TCID_50_ assay, serial dilutions of virus were incubated with Vero E6 cells in 96‐well plates induplicate. The cytopathic effect of each well was observed and recorded 60 h post‐infection. The titres of infectious viruses are expressed as log_10_ TCID_50_ mL^‐1^. For the plaque assay, a tenfold dilution of virus infected Vero E6 cells in 6‐well plates for 2 h. The virus inoculum was removed and the cells were supplemented with culture media. Cells were fixed 3 days after infection, and the plaques were visualised by staining with crystal violet.^[^ ^28^, ^59^ ^]^

### 5.4 Viral RNA extraction, reverse transcription, and real‐time quantitative polymerase chain reaction

Real‐time quantitative polymerase chain reaction (RT‐qPCR) was performed to assess the viral load and evaluate the ability of CAP to inactivate viruses in water. Viral RNA in the infectious supernatant was extracted using the Flying Shark Tissueand Cell RNA Kit (Nobelab Biotech, China). Reverse transcription was performed using the Hifair II 1st Strand cDNA Synthesis Kit (Yeasen Biotech, China) to obtain viral cDNA. RT‐qPCR was performed using a two‐stage TaqMan probe method. The probe and primer sequences are listed in (Table S1).

To measure gene copies, the PCR product was inserted into a vector to synthesise the standard plasmid. After determining the copy number (RuiBiotech, China), the plasmid was serially diluted (10^−3^–10^−9^) for RT‐qPCR analysis. A standard curve was generated based on the copy number and cycle threshold values.

### 5.5 Detection of viral attachment

SARS‐CoV‐2 trVLP and GX_P2V suspensions after CAP treatment for different durations were added to Caco2‐N and Vero E6 cells for 2 h at 4°C (for virus attachment) or 37°C (for virus attachment and entry). The cells were washed three times with phosphate‐buffered saline to remove free viruses, and collected for RT‐qPCR analysis.

### 5.6 Inactivation assay of pseudotyped SARS‐CoV‐2 variants

The SARS‐CoV‐2 pseudovirus was used to examine the inactivation effect of CAP on the SARS‐CoV‐2 variants. The pseudovirus was prepared using the vesicular stomatitis virus pseudovirus packaging system.^[^ ^60^ ^]^ CAP‐treated and untreated samples (100 μL) were incubated with BHK‐21 cells. After culturing for 24 h, the relative light unit (RLU) of the pseudovirus was determined by chemiluminescence detection using a microplate reader (BioTek, H1). The viral inactivation effect was calculated as follows:

$$\begin{array}{ccc} & & \mathrm{Inhibition}\left(\%\right)\\ & & \;=1-\frac{\mathrm{RLU}(\mathrm{CAP}-\mathrm{treated})-\mathrm{RLU}(\mathrm{blank})}{\mathrm{RLU}(\mathrm{CAP}-\mathrm{untreated})-\mathrm{RLU}(\mathrm{blank})}\times 100\% \end{array}$$

### 5.7 Cytotoxicity assay

Vero E6, Caco2‐N, and Huh7 cells were cultured in a culture medium which was pre‐exposed to CAP for various durations. After 48 h of incubation, the cells were treated with resazurin (Promega) for 2 h and the absorbance was measured at 570 nm. The cytotoxicity was calculated as follows:^[^ ^61^ ^]^

$$\mathrm{Inhibition}\left(\%\right)=1-\left(OD_{\mathrm{CAP}-\mathrm{treated}}/OD_{\mathrm{control}}\right)\times 100\%$$

### 5.8 Western blotting assay

Western blotting was performed to detect the production of GX_P2V nucleocapsid (N) protein after CAP treatment. Samples with the same protein concentration were loaded onto an SDS‐PAGE gel and transferred to a polyvinylidene fluoride membrane. The samples were blocked with 5% skim milk. Considering that the GX_P2V N protein shares 93.5% amino acid identity with the SARS‐CoV‐2 N protein, SARS‐CoV‐2 N protein antibody was used to detect the GX_P2V N protein.^[^ ^31^ ^]^ After washing out the blocking reagent, the SARS‐CoV‐2 N protein antibody (Genscript, USA) and glyceraldehyde 3‐phosphate dehydrogenase (GAPDH) antibody (Proteintech, USA) were incubated with the samples. After incubation, the antibody was washed, and HRP‐conjugated goat anti‐mouse immunoglobulin G (IgG) antibody was incubated with the samples. A chemiluminescent substrate (Thermo Scientific, USA) was used for imaging.

### 5.9 Enzyme‐linked immunosorbent assay

The receptor‐binding domain (RBD) is the key domain for the cellular entry of SARS‐CoV‐2. An ELISA was used to investigate the effect of CAP on the RBD activity of GX_P2V and SARS‐CoV‐2.^[^ ^26^ ^]^ The angiotensin‐converting enzyme 2 (ACE2) binding ability of the CAP‐treated viral suspensions was measured using a SARS‐CoV‐2 spike Protein RBD ELISA Kit (Beyotime, China). In addition, the N protein was detected using the SARS‐CoV‐2 Nucleoprotein/NP ELISA Kit (Solarbio, China).

### 5.10 Measurement of reactive oxygen and nitrogen species in cold atmospheric plasma

A hydrogen peroxide kit (HKM 0–25 mg L^‐1^, China) was used to measure the concentration of hydrogen peroxide. The measurement of ozone (O_3_) was performed using an ozone kit (HKM 0.05–1 mg L^‐1^, China). CAP‐generated reactive oxygen and nitrogen species (RONS) in the liquid phase were measured using various assay kits. A superoxide anion detection kit (Source Leaf Biology, China) was used to detect superoxide anion radicals (O_2_^−^) in the solution. The peroxynitrite anion (ONOO^−^) in the liquid samples was detected using an ONOO^–^ detection kit (enzyme‐free, MM‐0798M1, China).

### 5.11 Scavenger addition assay

To further verify the role of short‐lived and long‐lived substances generated by plasma in virus inactivation, 10 mm sodium pyruvate, 100 μm uric acid, 500 U mL^‐1^ superoxide dismutase (SOD), and 1 mm ebselen were used to quench H_2_O_2_, O_3_, O_2_^−^, and ONOO^−^, respectively. The scavenger was added to the viral suspension before CAP treatment. A viral suspension without scavengers was used as the control. All samples were subjected to CAP treatment for 60 s. In addition, titres of the virus suspension treated with scavengers were measured to exclude the effect of scavengers on viruses and cells.

### 5.12 Statistical analyses

All experiments were repeated three times with similar results. Statistical analysis was performed using the t‐test for two groups (GraphPad Prism8). The date was considered statistically significant when *p  <  0.05, **p  <  0.01, ***p  <  0.001, ****p  <  0.0001; ns means not significant.

## AUTHOR CONTRIBUTIONS

Ruixue Wang, Huahao Fan and Yigang Tong developed the concept. Ruixue Wang, Huahao Fan, Hongbo Qin and Hengju Qiu designed the research scheme. Hongbo Qin performed the virus and protein inactivation test. Hengju Qiu performed the plasma characterization and radical measurement assay. Ke Liu and Bixia Hong prepared the pseudovirus. Hongbo Qin, Hengju Qiu analyzed the data. Ruixue Wang, Hengju Qiu, Yuchen Liu and Chun Li established the plasma generator. Hongbo Qin, Hengju Qiu drafted the manuscript. Eric Robert, Huahao Fan., Ruixue Wang and Yigang Tong provided important experimental insights.

## CONFLICT OF INTERESTS STATEMENT

The Authors declare no conflicts of interest.

## Supporting information

Supporting Information

## Data Availability

All data needed to evaluate the conclusions in the paper are present in the paper and/or the Supplementary Materials.
