# Supplementary material for: Cold atmospheric plasma can effectively disinfect SARS‐CoV‐2 in the wastewater
Source: Exploration (Beijing). 2023 Nov 30;4(3):20230012. doi: 10.1002/EXP.20230012 (PMC11189572; doi:10.1002/EXP.20230012)
Supplement: Supplementary file 1 — Supporting Information [file EXP2-4-20230012-s001.docx]

**Supplementary information (SI)**

**Cold Atmospheric Plasma can effectively disinfect SARS-CoV-2 in the Wastewater**

Hongbo Qin^1,2*^, Hengju Qiu^1*^, Ke Liu^2*^, Bixia Hong^2*^, Yuchen Liu^1^, Chun Li^1^, Mengzhe Li^2^, Xiaoping An^2^, Lihua Song^2^, Eric Robert^3^, Yigang Tong^2,4#^, Huahao Fan^2#^, Ruixue Wang^1#^

1 College of Mechanical and Electrical Engineering, Beijing University of Chemical Technology, Beijing 100029, China;

2 College of Life Science and Technology, Beijing University of Chemical Technology, Beijing 100029, China;

3 University of Orléans, 3 boulevard Jean Jaurès, 45000 Orléans, France;

4 Beijing Advanced Innovation Center for Soft Matter Science and Engineering, Beijing University of Chemical Technology, Beijing 100029, China.

*These authors contributed equally.

^#^Correspondence: Yigang Tong: [tong.yigang@gmail.com](mailto:tong.yigang@gmail.com); Huahao Fan: [fanhuahao@mail.buct.edu.cn;](mailto:fanhuahao@mail.buct.edu.cn;) Ruixue Wang: wrx@mail.buct.edu.cn.

**Table of Contents**

**Legends**

Supplementary Figure 1. Electrical parameters of CAP.

Supplementary Figure 2. Representative images of Vero E6 cells infected with GX_P2V (different treatments).

Supplementary Figure 3. The CAP exposure effect on coronavirus PEDV infection in a large volume of water.

Supplementary Table 1. Primers used in RT-qPCR.

**Figures**

**Supplementary Figure 1.** Electrical parameters of CAP. (A) Instantaneous power and energy waveforms of CAP generation device. (B) Voltage and current discharge waveforms.

**
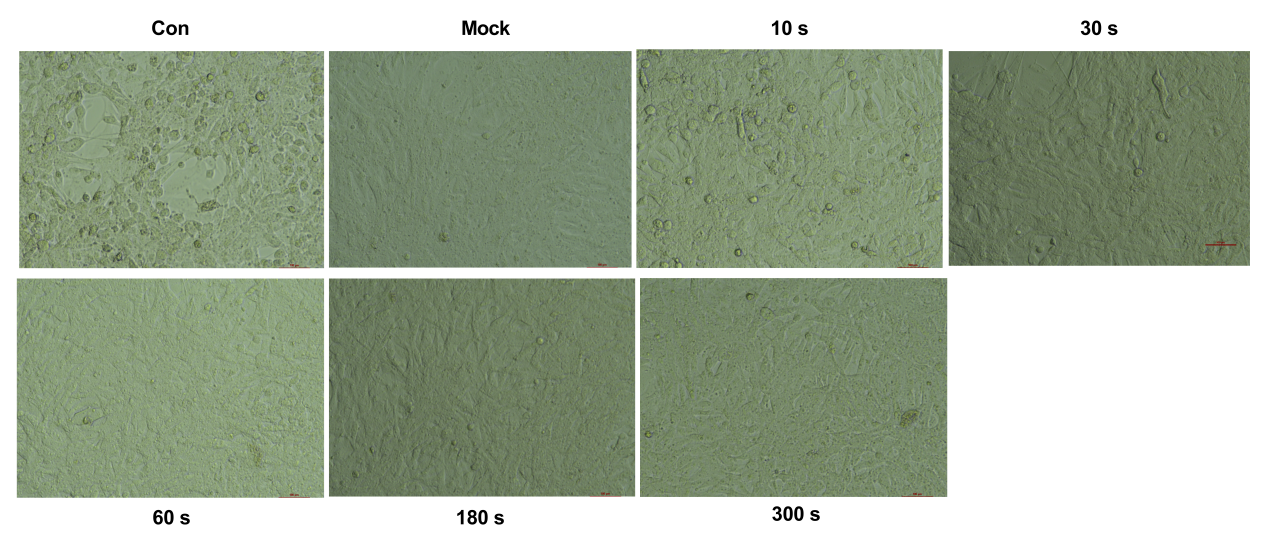
**

**Supplementary Figure 2.** Representative images of Vero E6 cells infected with GX_P2V (different treatments).

**
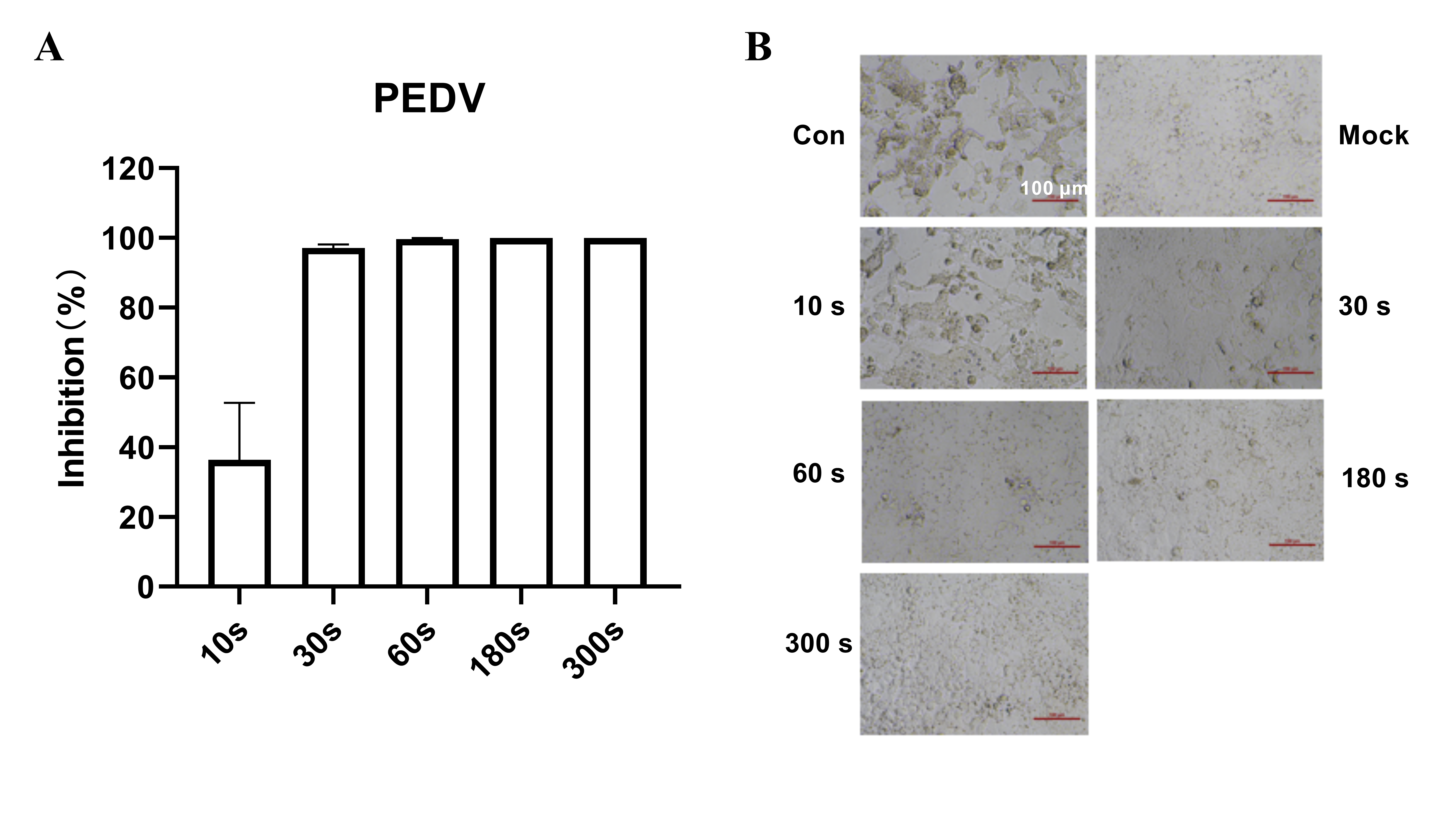
**

**Supplementary Figure 3.** The CAP exposure effect on coronavirus PEDV infection in a large volume of water. (A) Inhibition (%) of PEDV infection at various CAP exposure durations. (B) Representative images of PEDV infected Huh7 cells with different treatment.

**Supplementary Table 1.** Primers used in RT-qPCR

| Primers | Sequences |
| --- | --- |
| GX_P2V-F | 5’-GGTGATTGCCTTGGTGATATTG-3’ |
| GX_P2V-R | 5’-GCAAGTAGTGCAGAAGTGTATTG-3’ |
| GAPDH-F | 5’-AGCCTCAAGATCATCAGCAATG-3’ |
| GAPDH-R | 5’-ATGGACTGTGGTCATGAGTCCTT-3’ |
